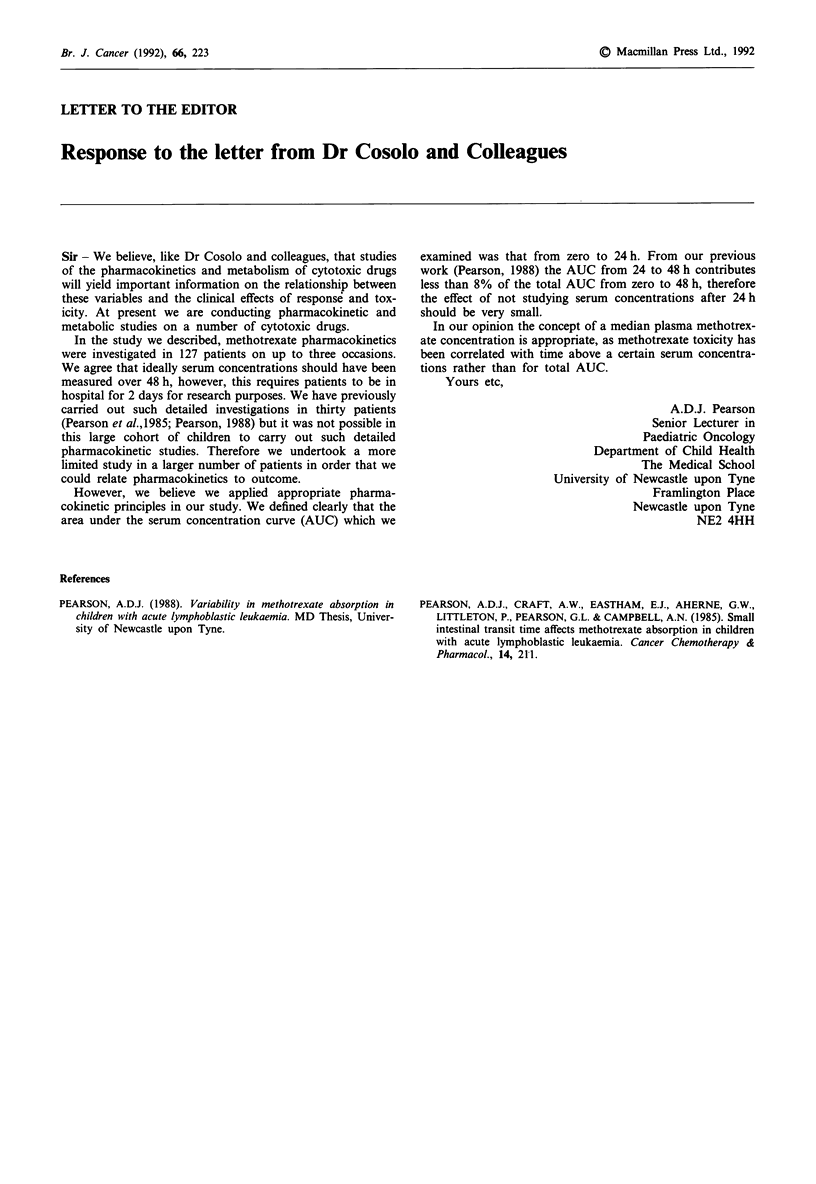# Response to the letter from Dr Cosolo and Colleagues

**Published:** 1992-07

**Authors:** A.D.J. Pearson


 © Macmillan Press Ltd., 1992

### LETTER TO THE EDITOR

### Response to the letter from Dr Cosolo and Colleagues

**Sir** – We believe, like Dr Cosolo and colleagues, that studies of the pharmacokinetics and metabolism of cytotoxic drugs will yield important information on the relationship between these variables and the clinical effects of response and toxicity. At present we are conducting pharmacokinetic and metabolic studies on a number of cytotoxic drugs.

In the study we described, methotrexate pharmacokinetics were investigated in 127 patients on up to three occasions. We agree that ideally serum concentrations should have been measured over 48 h, however, this requires patients to be in hospital for 2 days for research purposes. We have previously carried out such detailed investigations in thirty patients (Pearson *et al.*,1985; Pearson, 1988) but it was not possible in this large cohort of children to carry out such detailed pharmacokinetic studies. Therefore we undertook a more limited study in a larger number of patients in order that we could relate pharmacokinetics to outcome.

However, we believe we applied appropriate pharmacokinetic principles in our study. We defined clearly that the area under the serum concentration curve (AUC) which we examined was that from zero to 24 h. From our previous work (Pearson, 1988) the AUC from 24 to 48 h contributes less than 8% of the total AUC from zero to 48 h, therefore the effect of not studying serum concentrations after 24 h should be very small.

In our opinion the concept of a median plasma methotrexate concentration is appropriate, as methotrexate toxicity has been correlated with time above a certain serum concentrations rather than for total AUC.

Yours etc,

A.D.J. Pearson
Senior Lecturer in
Paediatric Oncology
Department of Child Health
The Medical School
University of Newcastle upon Tyne
Framlington Place
Newcastle upon Tyne
NE2 4HH

### References

PEARSON, A.D.J. (1988). *Variability in methotrexate absorption in children with acute lymphoblastic leukaemia.* MD Thesis, University of Newcastle upon Tyne.

PEARSON, A.D.J., CRAFT, A.W., EASTHAM, E.J., AHERNE, G.W., LITTLETON, P., PEARSON, G.L. & CAMPBELL, A.N. (1985). Small intestinal transit time affects methotrexate absorption in children with acute lymphoblastic leukaemia. *Cancer Chemotherapy & Pharmacol.*, **14,** 211.